# Editorial: Should We Treat Aging as a Disease? Academic, Pharmaceutical, Healthcare Policy, and Pension Fund Perspectives

**DOI:** 10.3389/fgene.2016.00017

**Published:** 2016-02-16

**Authors:** Alex Zhavoronkov, Alexey Moskalev

**Affiliations:** ^1^The Biogerontology Research FoundationOxford, UK; ^2^Insilico Medicine Inc.Baltimore, MD, USA; ^3^Laboratory of Regenerative Medicine, Federal Research and Clinical Centre of Pediatric Hematology, Oncology and ImmunologyMoscow, Russia; ^4^Radiation Ecology, Laboratory of Molecular Radiobiology and Gerontology, Institute of Biology of Komi Science Center of Ural Branch of RASSyktyvkar, Russia; ^5^Biological and Medical Physics Department, Moscow Institute of Physics and Technology (State University)Dolgoprudny, Russia

**Keywords:** biogerontology, longevity risk, aging disease, geroprotectors, WHO, ICD-11

The quest to increase healthy lifespan is becoming a pressing economic priority required to preserve the current standards of living. Rapidly increasing dependency ratios and unfunded social security and healthcare liabilities are an enormous and growing burden on the economies of developed countries (Zhavoronkov et al., 2012; Zhavoronkov, 2013). But the situation, if handled properly, is not hopeless; with advances in anti-aging treatments and preventative care, the negative economic impact of aging could be at very least reduced, while increases in productive longevity in developed countries could actually stimulate significant economic growth (Zhavoronkov and Litovchenko, 2013).

One of the impediments to industry transformation is the way aging is treated. While no doubt exists that aging is a complex multifactorial process leading to a progressive decline in function with no single cause or treatment (Zhavoronkov and Cantor, 2011; Moskalev et al., 2014), the issue of whether aging can be classified as a disease is widely debated by gerontologists, medical doctors, demographers, philosophers, policy makers, and the general public. This disagreement has until now hindered classification of aging as a disease and, consequently, the fitting of potential treatment options into established research, regulatory, insurance, and marketing frameworks.

By initiating a call for papers via a research topic with a descriptive “Should we Treat Aging as a Disease? Academic, pharmaceutical, healthcare policy, and pension fund perspectives” title in Frontiers in Genetics, we gathered the opinions of the many stakeholders including representatives of the pharmaceutical and biotechnology industries, demographers, and research scientists. None of the representatives of the pensions fund and insurance industries queried responded to the call, which can be explained by the general attitude toward aging and longevity in these industries (Zhavoronkov, 2015).

Some of the prominent biogerontologists provided comprehensive weighted responses explaining the dangers of separation of aging from disease and benefits of proactive preventative approaches that are likely to result from recognizing the pathological nature of aging. In spite of the many breakthroughs providing proof of concept for successful interventions in aging in model organisms, human progress has been surprisingly slow. One major cause of inaction is a widely held, but flawed, conceptual framework concerning the relationship between aging and disease that categorizes the former as “natural” and the latter as “abnormal” (Faragher). Gems concluded a comprehensive review of the many arguments for and against classifying aging as a disease with a definite and eloquent recommendation that calls for a complete quote: “We must draw aside the rosy veil of tradition and face aging for what it is, and in all its horror: the greatest disease of them all” (Gems).

Bulterijs et al. explained the many benefits of classifying aging a disease (Bulterijs), while Stambler provided a historical perspective arguing that acknowledging the possibility of successful intervention into the aging process, in other words treating aging as a curable disease, has been a long and highly respected tradition of biomedical thought (Stambler). Dubnikov and Cohen provided an overview of multiple theories of aging and recommended further research to understand the relationship between aging and disease (Dubnikov and Cohen).

Vaiserman proposed taking a systems-oriented approach looking at the plurality of genetic pathways and epigenetic mechanisms for identifying aging-modulating interventions (Vaiserman) complementing the signalome-wide approach for geroprotector screening (Zhavoronkov et al.). Zarling et al., proposed using nitroxide agents as possible drugs targeting age-related macular degeneration and other age-related diseases (Zarling et al.), and Luo et al. proposed a healthcare economics-driven model for development and adoption of companion diagnostics (Luo et al.).

Advocates for longevity research provided new survey data indicating that the majority (74.4%) of Americans are interested to live to 120 or longer if health was guaranteed, but only 57.4% wished to live that long if it wasn't (Donner et al.), contradicting previous surveys that used different approaches to surveying the general population and generally indicated negative attitudes toward increased longevity and longevity-boosting interventions (Duncan, 2012; Pew Research Center, 2013).

Many age-related diseases and genetic disorders share common pathways with normal aging (Tacutu et al., 2011; Makarev et al., 2014; Aliper et al., 2015). There is evidence indicating that longevity can be extended with a variety relatively non-toxic interventions (Moskalev et al., 2015) and there are multiple promising treatments in the pipeline. However, while there are many scientists and organizations providing arguments for and outlining the many economic and societal benefits of recognizing aging as a disease, there are few proposals describing concrete steps toward classifying aging as a disease. Moreover, many arguments lack realization that aging in the form of senility and senescence is already classified as a disease by some of the most influential agencies.

The main international agency responsible for disease classification is the World Health Organization (WHO), which maintains and publishes the International Statistical Classification of Diseases and Related Health Problems (ICD) since 1948. The 10th revision of the ICD, referred as ICD-10, was first published in 1992 (World Health Organization, 1992), and the 11th revision (ICD-11) is expected to be released in 2018 (http://www.who.int/classifications/icd/revision/timeline/en/).

WHO classifies aging as a disease in the ICD-10 with the “R54” code (World Health Organization, 1992). However, this code is generally regarded by the Global Burden of Disease (GBD) statisticians as a “garbage code” (Murray and Lopez, 1996; Lozano et al., 2013) and cannot be considered to be actionable. Actionable classification of aging as a disease may lead to more efficient allocation of resources by enabling funding bodies and other stakeholders to use quality-adjusted life years (QALYs) and healthy-years equivalent (HYE) as metrics when evaluating both research and clinical programs. In order to classify aging with an actionable code or set of codes linked to specific age-related diseases, authors propose an international task force to be organized to develop and communicate proposals to the WHO at the national and international levels (Zhavoronkov and Bhullar). We propose starting with reclassification of age-related muscle wasting or sarcopenia as a treatable medical condition, considering the number of interventions developed within the pharmaceutical industry and academia.

## Author contributions

AZ, AM organized the research topic and wrote the paper.

### Conflict of interest statement

AZ and AM are affiliated with research-oriented companies but declare no financial interest in this study. AZ and AM declare that presently they are terminally ill with aging.
